# Does generativity matter? A meta-analysis on individual work outcomes

**DOI:** 10.1007/s10433-022-00727-w

**Published:** 2022-09-19

**Authors:** Justyna Wiktorowicz, Izabela Warwas, Dariusz Turek, Iwa Kuchciak

**Affiliations:** 1 Department of Economic and Social Statistics, Faculty of Economics and Sociology, University of Lodz, 41 Rewolucji 1905 Street, 90-214 Lodz, Poland; 2 Department of Labour and Social Policy, Faculty of Economics and Sociology, University of Lodz, Lodz, Poland; 3 Institute of Enterprise, Collegium of Business Administration, Warsaw School of Economics, Warsaw, Poland; 4 Department of Banking, Faculty of Economics and Sociology, University of Lodz, Lodz, Poland

**Keywords:** Generativity, Work, Ageing, Generations, Work outcomes

## Abstract

Ongoing demographic changes and global population ageing require organisations to pay special attention to their employment policies. With working life extension and age management increasingly included in discussions about reactive versus proactive personnel policies, the term ‘generativity’ gains special importance as an approach to managing a generationally diverse workforce. Generativity can be understood as an attitude of openness towards the younger generations that focuses on exchanging values, knowledge, and experiences with them. It is a source of positive emotions and better social relationships, personal fulfilment, good energy, and aliveness. In the paper, generativity is discussed in the framework of two theories: the socio-emotional selectivity theory (SST) and successful ageing theory (SOC). The aim of this paper is to assess the relationship between generativity and individual work outcomes. We considered both in-role and extra-role outcomes analysed in the job context. Meta-analysis is conducted of studies that investigate generativity and its relationships with motivational outcomes (job satisfaction, engagement, work motivation, affective commitment, self-efficacy), cognitive outcomes (attitudes toward retirement, career success, self-control), personal outcomes (wellbeing, health, job strain), relational outcomes and extra-role behaviours (organisational citizenship behaviour and sustainable behaviour). The analysis examines 65 independent samples that included 30,540 individuals, and considers the role of three moderators—the cultural context, the measurement method and age. It demonstrates that generativity has significant and positive motivational, cognitive and extra-role behaviour outcomes for workers and that it improves their well-being.

## Introduction

Ageing is one of the primary challenges facing developed economies (Aisa et al. 2015) and one of the megatrends that affect workplaces (Linthorst and de Waal 2020; Klein et al. 2017). One way to reduce the negative consequences of ageing related to the growing old-age-dependency like the loss of responsibility, loss of role and loss of social contact is to stimulate the extending working life (Donovan and Blazer 2020). The increasing life expectancy means that we can work longer while maintaining our individual productivity (Weber and Lichinger 2020; Burtless 2013). Individual productivity will provide productivity at the organisational level if older workers are open to sharing their knowledge and skills with youngers. Individual productivity and self-efficacy contribute to creating perspective for further career and sense of success. As evidenced by the Eriksonian developmental theory (Erikson 1968, 1982), people in the 7th stage of their life (aged 40–65) are often concerned about the future and feel the need to guide younger people and contribute to the next generation.

McAdams and de St. Aubin (1992) and Mor-Barak (1995) were the first to place generativity in the organisational context. Generativity improves interpersonal relations, increases involvement and vitality, and motivates people to act and share their knowledge and experience with others (McAdams et al. 1993). It can be expected that older workers’ individual outcomes (such as job satisfaction, motivation, engagement, loyalty, trust etc.) will be associated with their generativity (Boselie 2010). Research in this area has already been conducted, but it was usually narrowed down to specific outcomes, analysed either only in a strictly professional context (in-role behaviour) or going beyond the professional role (extra-role behaviour, e.g. citizenship or sustainable behaviour). Such studies are often based on small samples, and meta-analyses that refer to these relations are rare. This paper fills this gap.

The aim of this paper is to assess the relationship between generativity and individual work outcomes. We considered both in-role and extra-role outcomes analysed in the job context.

This paper makes three important contributions. Firstly, it offers a cumulative perspective on the link between generativity and five types of individual outcomes. Secondly, the use of moderator variables (the cultural context, age and method of generativity measuring) provides a better insight into what circumstances cause generativity to have a stronger or weaker effect on individual outcomes less. Lastly, the empirical data collected for the paper allow a closer look at theories that explain the process and the effects of generativity.

## Generativity in the work context—theoretical and conceptual approach

The term generativity, introduced by Erikson (1963, 1982), is comprised of “cultural demands, inner desire, generated interest, belief, commitment, and personal narration to produce action” (Nuri 2017). It is defined as strips of experience that bring a feeling of energy and aliveness to people, and that also have the potential to produce more enduring expansive and transformative consequences (Carlsen and Dutton 2011).

Generativity contributes to a feeling of personal fulfilment and a sense of immortality among people approaching the end of their lives (Henry et al. 2015; Lang and Carstensen 2002). It is a force that “propels and motivates actions” and allows for the creation of an optimal space (Carmeli and Dothan 2017), in which new vistas are opened up, and resources are expanded on and cultivated (Carlsen and Dutton 2011). It enables people to develop “a new sense-making” (Bushe 2010) that offers possibilities, and provides them with the opportunity to generate, learn, and seek new things (Carmeli et al. 2016). It also taps the quality of relationships formed and cultivated between team members that injects more positive energy and aliveness (Carlsen and Dutton 2011).

Generativity is assumed to "improve" with age, as workers, parents, volunteers, and adults may experience and express concerns for the next generation (McAdams et al. 1993). Older people are a sort of a ‘conveyor belt’ that delivers valuable standards and values. Mor-Barak (1995) demonstrated that for older adults, work is an opportunity to share their knowledge and experiences, and to transmit ideas and values to the younger generations. Following Mor-Barak’s work, Dendinger et al. (2005) pointed to four reasons for work (social, personal, financial, and generative) and three reactions to bridge employment (satisfaction with work, attitude to retirement, and a sense of being effective at work). Generative behaviour can be significantly influenced by values, beliefs, and political views (Nuri 2017), as well as by neighbours, friends, and cultural and leisure activities (McAdams et al. 1993, 1997). A reflection of this approach can be found in works that emphasise that the workplace is not the only place where active, prosocial attitudes to others and productive efforts are born.

Age-related individual changes in generativity stress its significance in striving for well-being in old age, as generativity offers the opportunity to be productive in a social context (Hofer et al. 2014; Peterson and Klohnen 1995). Higher levels of generativity are associated with longevity, better physical functioning and the psychological well-being of older adults (Grossbaum and Bates 2002; Au et al. 2020; Pozzi et al. 2014). Relating to McAdams and de St. Aubin (1992), Krumm et al. (2013b) wrote that generativity may manifest itself in three ways: creation (e.g., producing something meaningful), retention (e.g., preserving something of worth), and offering (e.g. helping and passing on knowledge).

Because two lifespan theories—the selective optimization with compensation theory (SOC) and the socioemotional selectivity theory (SST)—allow to understanding, among other aspects, how people cope with gains and losses over their lifespan, we used them to analyse how generativity relates to individual outcomes. According to SOC theory (Baltes and Baltes 1990), three distinct behavioral strategies (selection, optimisation, compensation) lead to positive outcomes such as goal accomplishment, work ability, job performance, work engagement, and occupational well-being, because the combined use of these strategies helps individuals to optimally allocate their limited resource. An individual’s allocation of resources aimed at growth decreases with age, whereas the allocation of resources used for maintenance and the regulation of loss increases with age. In the socioemotional selectivity theory (SST) (Carstensen et al. 1999) the core construct is the future time perspective (FTP). The perception of time plays a fundamental role in the selection and pursuit of goals, in particular goals related to knowledge acquisition, social contact, and emotional experience (Henry et al. 2017). SST theory holds that priorities shift from striving for developmental goals to the pursuit of socioemotional or generativity goals (Kooij et al. 2013a). The shift allows people to learn to age and increases the significance of goals that are achievable in the short term. They become more appreciative of close social relations and invest more in their quality. They also feel a stronger urge to “leave a trace” (generativity goals), and social activity and a sense of belonging to their milieu give them a range of benefits.

Likewise, Karlsen et al. (2022) note, selection, optimisation, and compensation strategies were encountered at all of the organizational levels (except compensation at the leadership level). Nevertheless, despite the theoretical argumentation only a few studies have attempted to include other organizational levels than the individual when working with the SOC model. Thus, searching and selecting generativity papers from the last twenty years we found that also generativity effects were analysed mainly from the individual perspective. That has set our approach to analysis and classification of generative outcomes in the workplace. Since we analysed generativity effects on the individual level in the job context, we concentrated on workers’ perspective. And since we considered benefits which workers and consequently their employers could derive from generative attitudes and behaviours, the human resources effects are key in our study.

In the literature, human resources effects are studied from different perspectives, usually in terms of output, effectiveness, efficiency, outcomes, or even organisational performance measured by financial aspects (Kaplan and Norton 1993). Guest (1997) proposed the concept of outcomes, which Dyer and Reeves (1995) divided into three categories: (1) financial outcomes, (2) organisational outcomes, and (3) HR outcomes. Taking into consideration generative (i.e. human/ workers) context, in papers analysed within our metaanalysis only the last group–HR outcomes (i.e. employees’ attitudes and behaviour) are included.

**Fig. 1:**
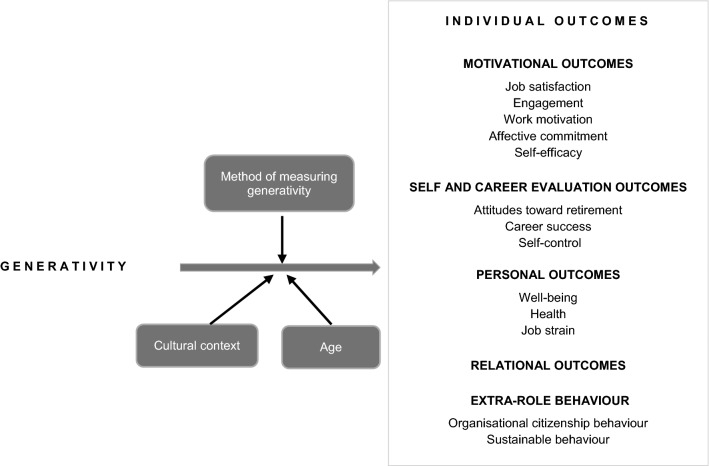
Conceptual model of the relationships between generativity and individual work outcomes

In the author's model, we divided outcomes on individual level into five groups. Motivational aspects like emotions and feelings in our model are represented by: job satisfaction, engagement, work motivation, affective commitment, and self-efficacy. Employees’ self-assessments of their workplace situation (the self and career evaluation component) are associated with their attitudes toward retirement, career success, and self-control. From the job perspective, relational outcomes (interpersonal relations) and personal outcomes such as well-being and health and job strain are also important. Using a wider, interdisciplinary perspective of individual outcomes, we also considered behaviours beyond the formal contract with the organisation, i.e., extra-role behaviours (van Dyne et al. 1995), and more specifically, organisational citizenship behaviours and green behaviours. We realize that like Guest (1997) mentioned, researchers might wish to link employee perceptions to their behaviour, to individual or group-level performance outcomes which affect unit performance and thus to company profits. We also agree that HR policies, practices and interventions directly influence HRM outcomes (see, for instance, Boselie 2010; Collins et al. 2005). In analysing the relationships between generativity and individual outcomes, we also considered three moderators—the method of measuring generativity, age and the cultural context (Fig. 1).

We assume that the five categories of individual outcomes provide numerous benefits to older workers, but are also beneficial to organizations, teams, or colleagues of all ages.

## Generativity and individual outcomes

### Generativity and motivational outcomes

Motivational outcomes (such as job satisfaction, engagement, work motivation, affective commitment, and self-efficacy) are thought to be positive and most significantly related to generativity. Job satisfaction is an attitude towards one’s job that reflects the evaluation of prior work experience (Krumm et al. 2013a), and it has an important effect on retirement decisions (Kosloski et al. 2001). Job satisfaction can reportedly be increased by generativity motivation (Stamov-Roßnagel and Biemann 2012; Dendinger et al. 2005) and that among the various reasons for working—social, personal, financial, and generative—only the last one is a reliable predictor of job satisfaction (Dendinger et al. 2005). This is in line with socioemotional selectivity theory (SST)—aging goes along with shifts in individuals’ motives and goals, so younger and older employees derive satisfaction from different aspects of their jobs (Kooij et al. 2011).

Clark and Arnold (2008) analysed the associations between generativity concern (wishes, values, and self-perceptions), generativity commitment (goals, plans, and intentions), generativity action (actual behaviour), and job satisfaction. They showed that in each approach generativity was significantly and positively related to job satisfaction. Millová and Blatný (2018) also confirmed the positive correlation between generativity concern and job satisfaction. Shilo-Levin et al. (2021) went further and confirmed that a high sense of meaning at work is related to different positive organisational outcomes, such as organisational commitment, engagement and performance (Geldenhuys et al. 2014) and work satisfaction (Duffy et al. 2015).

Mansour and Tremblay (2019) demonstrated that the use of generativity, as well as knowledge transfer to the next generation, can offer the resources lead to more job satisfaction. Meanwhile, considering the Pratt and Lawford (2014) research, it is worth to note that generative concern was predictive of higher levels of subsequent civic involvement in early adulthood. At work, generativity may help strengthen youth goals of social responsibility, which in turn were linked to greater subsequent job satisfaction. On the other hand, Pundt et al. (2015) concluded that for people who work after retirement, having relationships, a strong self-belief, and the desire for freedom in decision making and the need to pass on knowledge and experiences might contribute more to satisfaction with life than job satisfaction.

Intrinsically rewarding jobs might motivate individuals in midlife to feel they are helping to establish and guide the next generation. Chen et al. (2019) and Krahn et al. (2021) proved that those who feel intrinsically rewarded from working in jobs that require extensive decision-making and provide a sense of accomplishment will also feel a greater sense of generativity.

De Lange et al. (2010), Kooij et al. (2013a), and Burmeister et al. (2021) found that generativity is positively related to work engagement. Employees feel more connected and closer to each other due to the unique nature of intergenerational contact (Burmeister et al. 2021). As Ding and Schuett (2020) found generativity also has a positive effect on volunteers—the higher the generativity level, the higher their motivations, satisfaction, and commitment.

Commitment and engagement can be related to self-efficacy. Self-efficacy (Bandura 1977) denotes the belief in one’s capability to perform a specific task. Self-efficacy is a fundamental ability to cope, perform, and be successful (Judge and Bono 2001; Fletcher et al. 1992). It can also be belief in one’s ability to learn and use new job skills, meet the challenge of change, and remain productive, which is of special importance for older workers (Dendinger et al. 2005). It is widely believed that older workers do not perform as well as younger workers in jobs that require the retrieval and processing of significant amounts of information, and that their awareness of their reduced performance capability may and decrease self-efficacy (Griffiths 2000). Also, older workers’ feeling that they are no longer able to optimally engage in work activities may have an adverse impact on their job satisfaction and involvement (Clark and Arnold 2008). On the other hand, workplace performance may benefit from increasing the share of older workers for several reasons. According to human capital theory, individuals accumulate human capital over the life cycle and become more productive as they learn from their work experience. This experience enables workers to become more proficient at job tasks, learn new skills, and assimilate working routines, thereby becoming more productive with age in various settings (Bryson et al. 2020). Moreover, organizations need to find ways to create work environments that satisfy the specific needs of employees of different age groups (Bal and De Lange 2015). One of the prerequisites for worker efficiency is to modify the nature of their work to best fit their capabilities (which include their state of health and predispositions). Such modifications will allow an enterprise to make better use of the worker’s potential and increase their motivation to work (Kollmann et al. 2020).

The previous deliberations were used to formulate the hypothesis:

#### H1

 Generativity is positively related to the motivational outcomes of workers.

### Generativity and self and career evaluation outcomes

Among the self and career evaluation outcomes, attitudes toward retirement, career success and self-control are important. In the analysed papers, an employee’s attitude to retirement was understood as their willingness to retire or extend their working life. This HR outcome is important because it may impair organisational performance directly (employees working below their capacity) or indirectly (by increasing turnover intension) (Marescaux et al. 2012). Older employees have different attitudes to retirement—some continue to work after reaching the statutory retirement age, some retire completely, while others choose bridge employment (a part-time job, self-employment, or a temporary job and drawing a pension) (Kim and Feldman 2000). Employees’ attitudes to retirement have been studied, for example, by Dendinger et al. (2005), Mansour and Tremblay (2019), Mor-Barak (1995) and Zhan et al. (2015). Schermuly et al. (2014) introduced in their research also the desired retirement age and proved that it is beneficial for organisations, and the generativity control variable has the positive effect.

A positive relationship between generativity and attitudes to continuing work (bridge employment) was reported by many authors (Dendinger et al. 2005; von Bonsdorff et al. 2009; Zhan et al. 2015). Micheel (2019) concluded that people scoring higher on the generativity scale were less inclined not to work at all than to take paid or unpaid jobs. Hess et al. (2020) concluded that generativity motives are less important than financial one—people which main motive is knowledge sharing state that they want to work significantly fewer hours and days. In the study by Brougham and Walsh (2009), both early and late retirees believed that generativity goals were more attainable for those who continued to work than for those who chose retirement. Mansour and Tremblay (2019) concluded that retirement preparation (also understood as remaining time at work—Burmeister et al. (2021)) was unrelated to generativity. Arnold and Clark (2016), in turn, showed that a forward momentum career orientation was positively correlated with generativity and that maintaining a career outlook may stifle generativity rather than encourage it.

Career success is typically interpreted as changes in the positions occupied by individuals and changes in their personal characteristics related to the former. In Erikson's understanding of generativity (Erikson 1968), people are most concerned with becoming a supervisor at the mid-life career stage, and then they become increasingly preoccupied with their legacy and aware of their need to be needed (Clark and Arnold 2008; Kanfer and Ackerman 2004). This relationship may vary with the form of generativity. Clark and Arnold relate success to interpersonal and societal generativity, but only in the domain of action (Clark and Arnold 2008). In a later study (Arnold and Clark 2016), they concluded that the forward momentum strategy was positively correlated with generativity and generativity depended on whether workers perceive their careers as advancing or in stationary and less expansive terms. In the study by van der Heijden et al. (2008), career satisfaction was found to be significantly correlated with generativity, but only for career-minded people (i.e., other than the self-employed and people in bridge employment), while Templer et al. (2010) found this link to be significant also for the bridge employed. Zacher et al. (2011) found a positive relationship between leaders’ generativity and how their followers perceive their late-career effectiveness.

Self-control can be defined in the job context as the ability to set one’s own goals, to present them convincingly, and to make decisions, as well as the ability to constructively deal with opposition and conflicts (Rachlin 2004). There is research evidence pointing to a positive effect between self-control and generativity (Sanders and McCready 2010), effective problem-solving in the workplace (Isen and Reeve, 2005) or increased motivation (Muraven and Slessareva 2003). Vötter and Schnell (2019), who examined the impact of generativity on subjective well-being via a sense of meaning, proposed that self-control might be a personality factor that differentiates the respondent groups.

Based on the previous deliberations, the following hypothesis was formulated:

#### H2

Generativity is positively related to workers’ self and career evaluation outcomes.

### Generativity and personal factors

A variety of personal factors are important for work attitude and behaviour; therefore, we used well-being and health (physical and mental, in which stress) and examined them more closely from the occupational perspective. We adopted the definition of occupational well-being proposed by Warr (1987), who described it as the overall quality of an employee’s experience and functioning at work characterised by four dimensions—affective well-being, aspiration, autonomy, and competence. From the psychological perspective, well-being can be defined as a multifaceted construct with hedonic and eudaimonic dimensions (Ryan and Deci 2001). Most studies that deal with the hedonic dimension focus on assessing subjective well-being (SWB), interpreted as life satisfaction and the presence of a positive mood and the absence of a negative mood, which altogether represent happiness (Diener and Lucas 1999; Bonsang and Klein 2012). The eudaimonic perspective is related to measuring psychological well-being (PWB), which taps autonomy, personal growth, self-acceptance, life satisfaction, life purpose, mastery, and positive relatedness (Ryff 1989).

Erikson’s theory prompted researchers to test generativity as a mechanism that influences well-being, but because well-being was analysed from three different perspectives (hedonic, eudaimonic, and hedonic-eudaimonic), it was first necessary to integrate them into a coherent theory of human well-being by setting them in a cross-cultural framework of happiness (Delle Fave et al. 2011). Some authors then concluded that life satisfaction was a key aspect of well-being (Hofer et al. 2008; Pund et al. 2015), while many others presented evidence that generativity was significantly associated with life satisfaction (Hofer et al. 2008; Huta and Zuroff 2007; Karacan 2014; MacDermid et al. 1997; McAdams et al. 1993; Pundt et al. 2015; Shahen et al. 2019; Shilo-Levin et al. 2021; De Haan and MascDermid 1994).

Generative concern is indicated as being relevant to both the hedonic and eudaimonic conceptualisations of well-being (Grossbaum and Bates 2002), even in retirement (Spirling 2019; Serrat et al. 2018; Villar et al. 2013). In particular, mentoring investments positively predict generativity achievement in retirement (Chan and Nakamura 2015). Studies in the work context show that subjective well-being has a mediating effect on the relationship between job burnout and generativity concern (Lan 2019), while job burnout had a significant negative predictive effect on both subjective well-being and generativity concern (Lan et al. 2021).

Another important personal outcome of generativity is health. There is solid research evidence that poor health and the related problems that affect employability are very strongly associated with intentions to retire (see for example van Droogenbroeck and Spruyt 2014; Mutambudzi and Henkens 2020). In line with job demand-control theory (van Beurden et al. 2020), some studies negatively equate health outcomes with job stress and burnout (Jackson et al. 2014; Kilroy et al. 2016).

According to the lifespan theories (the SOC and the SST), specific age-related losses in biological potential cause older workers to change their goal focus. The SOC holds that as older workers experience losses in subjective general health, they shift their resources away from growth towards maintenance and regulation of loss. However, Kooij et al. (2013b) found that subjective general health does not mediate a negative relationship between age and growth motivations and a positive relationship between age and security motivations. Kooij and van de Voorde (2011) additionally established that changes in subjective general health are related to generativity and development motivations, as posited by the SST theory. Grossman and Gruenewald (2017) and Landes et al. (2014) demonstrated that generativity buffers the psychological effects of exposure to stress. Kooij et al. (2013b) found that generativity motivations increased with age, but only among university workers (in contrast to health workers). In the studies by Newton et al. (2020) and de Lange et al. (2010), the relationship between generativity and employees’ health was not significant, but such findings are rarely reported.

Based on the above, the following hypothesis was formulated:

#### H3

Generativity is positively related to workers’ personal outcomes.

### Generativity and relational outcomes

High-quality relationships in the workplace have a significant effect on learning behaviours that help organisations attain their goals (Cameron and Caza 2004). Generative women are characterised as having greater empathy, responsibility, self-control, tolerance, and well-being (Peterson and Klohnen 1995). Interpersonal relationships are considered in relation to generativity in two potentially competing life contexts, work and non-work, both of which are examined in terms of quantity, quality, duration (Lang and Carstensen 2002). Because generativity involves passing on knowledge and skills to the younger generation, researchers also include the concept of age bias in their analysis. Henry et al. (2015) found interactions between employee age and opportunities for generativity and development to predict intergenerational contact quality that is thought to negatively affect turnover intentions. They pointed out that the relationship between opportunities for generativity and intergenerational contact quality is positive among older workers and non-significant for young workers. However, Burmeister et al. (2021) came to the opposite conclusions. They found that intergenerational contact has the potential to motivate and engage both older and younger employees, and generative motive is positively related to intergenerational contact as well as the sense of belonging. Similarly, Fasbender et al. (2021) found that generativity striving was equally important for older and younger employees. Additionally, the moderation effect of the generativity motive on the relationship between intergenerational contact and a sense of belonging is not significant (Burmeister et al. 2021).

Based on the above, the following hypothesis was formulated:

#### H4

Generativity is positively related to workers’ relative outcomes.

### Generativity and extra-role behaviour outcomes

The specificity of generativity, and especially its extra-occupational context, encourages a broader look at organisational behaviour. When analysing organisational processes, apart from examining the professional activity inscribed in professional duties (in-role behaviour), it is possible to focus on behaviours that go beyond the formal contract with the organisation. Behaviours related to initiative, pro-sociality, pro-environmentalism, or caring for company resources are called organisational citizenship behaviours (Organ et al. 2006), extra-role behaviour (van Dyne et al. 1995), or prosocial organisational behaviour (Brief and Motowidlo 1986). However, they are most often a "discrete" form of employee activity, not taken into account by the formal motivation system, and they most often translate into positive consequences for the organisation. Krumm et al. (2013b) noted that generativity values refer to non-instrumental aspects of work, like enjoyment or social contact, and, thus, are likely to facilitate organisational citizenship behaviour (Ryan 2002). As Wells et al. (2016) confirm generativity is also positively related to green behaviours.

Based on the above, the following hypothesis was formulated:

#### H5

Generativity is positively related to extra-role behaviour outcomes.

## Potential moderators

### Generativity measures

The most popular generativity measure is the Loyola Generativity Scale (LGS), which assesses individual differences in generative concern (McAdams and de St Aubin 1992). This 20-item scale is usually used in the non-job context analysis. In the job context, the Meaning of Work Scale (MWS) is often applied. This method was developed by Mor-Barak (1995) to analyse the meaning of work of older adults. This 16-item scale allows to assess four factors—social, personal, financial, and generative. The generative factor is understood as teaching and training, and passing knowledge and skills to the younger generation. Based on the MWS, Kooij and van de Voorde (2011) conceptualized the generativity motives scale (Generative Striving–GS, 3-items).

Additionally, in a few single papers, other methods were proposed: the Q-Set Generativity Measure (Peterson and Klohnen 1995), the Role-Specific Generativity Scale, developed by Baruch et al. (1983), the Generative Behaviour Checklist (GBC) (McAdams and de St. Aubin 1992), the Munster Work Value Measure (MWVN) (Krumm et al. 2013b), the Thematic Apperception Test (TAT) is aimed at understanding people's intrinsic motives (or inner desires) to act in generative ways (Peterson and Stewart 1996), and a scale of leadership generativity (Zacher et al. 2011).

### Cultural context

Alexander et al. (1991) concluded that generativity is not a universal psychological principle but a cultural construct, so it should be analysed as a product of culture. Meanwhile, Rubinstein et al. (2015) suggested that to understand generativity and its outcomes, cultural approaches need to be taken into account. Some authors have implied that cultural differences may lead to differential relations between generativity and individual outcomes (Hofer et al. 2008, 2014; Kruse and Schmitt 2012). In this paper the geographical location of a study was taken into account. Because the availability of studies from outside Europe and North America was limited, preventing us from making specific country comparisons, we divided countries into three groups: North America and Australia, Europe, and Asia. The few non-European and non-North American samples included in our database came from countries deemed more collectivistic than European countries and the USA (e.g., China). In collectivistic cultures, intergenerational relationships are not only closer but also enshrined in cultural norms that require older workers to be respected and treated as mentors (Ghosh and Chaudhuri 2009). Consequently, employees in such cultures may be more focused on the relational and personal aspects of work. On the other hand, in more individualistic countries, such as the UK and the USA, employees may attach greater value to independence and focus more on the emotional aspects of work and workers’ self and career evaluation; then, generativity may intensify their attitudes and beliefs.

### Age differentiations

Theoretical evidence shows not only that age-related increase in generativity (Erikson 1963) but also that age of employee’ differentiates theirs attitudes towards work (Barnes-Farrell and Matthews 2007). Convincing empirical evidence in support of this thesis was provided by a meta-analysis by Ng and Feldman (2010). Using the criterion of dividing older workers by age 55 + found, among others, in the European Commission domains (EU 2016), but also in the analysed articles (e.g. Sanders and McCready 2010), we divided the study populations into age < 55 and age > 55. We also assume that there are differences between individual work outcomes among workers under and over 55.

## Methods

### Literature search and inclusion criteria

In order to select empirical studies on generativity outcomes for our analysis, two approaches were applied. The first approach involved searching through databases such as the Web of Science (SSCI), EBSCO, ABI/INFORM, ERIC, PsycINFO, Google Scholar, and Scopus, and databases that contain the results of various studies (e.g., metaBUS) for abstracts, keywords, and titles with the word ‘generativity’. The next step involved analysing all identified articles (between 2000 and 2020), which were published mainly in the following journals: *Int J Hum Resour Manag, Int J Manpow*, *J Manag, Work Aging Retire, Int J Aging Hum Dev*. Information about this meta-analysis was posted to the listservs of the Human Resources and Organisational Behaviour Division at the Academy of Management, and to researchers affiliated with ResearchGate.

Our meta-analysis focused on four categories of studies. The first included doctoral dissertations, book chapters, and journal articles about generativity, especially reliable because rigorous peer reviews (Siddaway et al. 2019). The second comprised empirical articles that measure generativity (conceptual articles were excluded) available in English to avoid construct ambiguity and interpretation errors. The third category contained studies conducted in a work context (similar to Doerwald et al. 2021). The fourth category was formed by separating works where variables were conceptualised as the consequences or moderators of generativity. Documents were excluded if the relevant statistics were not reported and the authors did not respond to, or were unable to fulfil, our request for such data. Lastly, we established a condition that generativity correlations should be reported at the individual level rather than at the group or organisational level. After removing studies that did not have the variables necessary to test our hypotheses, 65 independent samples were included in the final meta-analysis.

### Coding procedure

Most of the selected studies reported the effect size data using the *r*-Pearson correlation coefficient. When other metrics were used, they were converted to *r* using appropriate formulas. Studies utilising other multivariate analysis techniques, such as logistic regression, factor analysis, or multivariate analysis of variance, were excluded from the analysis unless a correlation matrix was provided or was obtainable from the authors. To preserve the independence of the samples, for each relationship studied, only one effect size was included from each sample. When a study reported data for multiple, independent samples, the samples were separately included in the analysis (Hunter and Schmidt 2004; Rudolph et al. 2021; Steel et al. 2021).

We assigned variables to the five categories of individual outcomes (Fig. 1). All four authors reached agreement on the categorisation, as shown by a high inter-rater agreement (Cohen’s kappa = 0.93).

The moderator variables were coded for three categories: the cultural context (Europe, North America and Asia), the measurement method (LGS, Mor-Barak’s MWS, GS and others) and age differentiations (below 55 age, over 55 age). Cohen’s kappa (1988) for all studies was 1.00. In the process of coding research into categories, we included these categories represented by a minimum of two independent samples (k ≥ 2). If there were at least two samples, we tried to meaningfully group constructs before excluding them. Table 1 provides an overview of the construct groupings.

**Table 1:** Construct groupings

General construct	Synthetic construct	Operationalisation
Motivational	Job satisfaction	Satisfaction with current work situation General work satisfaction Job satisfaction
	Work motivation	Work motivation Intrinsic motivation Meaningful work
	Affective commitment	Affective commitment
	Self-efficacy	Self-efficacy
	Engagement	Work engagement Extra effort Job involvement
Self and career evaluation	Attitudes toward retirement	Remaining time at work Desired retirement age Retirement attitudes Retirement preparation
	Career success	Career success
	Self-control	Self-control
Personal	Wellbeing	Life satisfaction General wellbeing
	Job strain	Stress Burnout
	Health	Health
Relational	Relational	Sense of belonging Intergenerational contact Networks Social satisfaction Knowledge sharing
Extra role behaviour	Extra role behaviour	Organisational citizenship behaviour Employee water and energy saving behaviour

### Meta-analysis procedures

Like other authors (Borenstein et al. 2011; Rothstein et al. 2002; Rudolph et al. 2021; Steel et al. 2021), we based our meta-analysis on the random-effects models, which assume that effect sizes in a population may vary, and that they can be influenced by factors other than sampling errors (Borenstein et al. 2011; Rudolph et al. 2021). The mean weighted correlations were calculated using the inverse of the variance components and accounting for both sampling errors and variation between studies, which allowed more equal weights between the studies to be obtained than if we used a fixed-effect model. Correlations corrected for attenuation were also calculated. The correlations were individually corrected for measurement errors in both predictor and outcome variables according to the Hunter and Schmidt meta-analysis method (Hunter and Schmidt 2004).

We determined the numbers of independent effect sizes (*k*), the sample size (*n*), mean correlation (*r*), sample size weighted correlation ($\overline{r}$), the 95% confidence interval for the mean effect (95% CI), the 95% credibility interval (95% CR), and the 95% prediction interval (95% PI). An effect size was regarded as significant if the confidence interval did not include zero.

We also calculated three statistics to quantify heterogeneity, i.e., the weighted sum of squares and its associated *p*-value (the *Q* statistic, which shows the relationship between the variance of the studies and individual standard errors), the *I*^*2*^ indicator, which measures the internal inconsistency of individual studies, and *T*^*2*^—the variance of the true effect sizes (Steel et al. 2021). Low *I*^*2*^ indicates a low probability that the studies are heterogeneous. To assess the heterogeneity of effect sizes, we calculated the sampling error variance. We assumed homogeneity when 75% or more of the observed variance could be explained by sampling error variance (Hunter and Schmidt 2004). High heterogeneity within a group suggests the presence of moderators. The moderator analysis was performed using Hunter and Schmidt’s (2004) approach (*z*-test). Categorial moderators (as in this study) were subjected to a meta-regression analysis using a random effects model with full maximum likelihood estimation.

We complemented the meta-analysis by a meta-regression, and we used a weighted least square regression with the random effect model (recommended for heterogeneity of effect sizes).

## Results

### The relationship between generativity and individual outcomes

The inclusion criteria resulted in a total of 65 independent samples (*k*) that included 30,540 individuals (*n*). The studies selected for analysis are denoted by an asterisk in the References. Many of the samples contained data on more than one relationship. A summary of the meta-analysis results is presented in Table 2. According to the effect-size benchmarks recommended by Steel et al. (2021), corrected correlations ranging from 0 to 0.10 were classified as small, 0.18 as medium, and those greater than 0.32 as large.

**Table 2:** Meta-analysis results for the relationships between generativity and outcomes

Variable	K	N	*r*	$\overline{r}$	95% CI	95% CR	95% PI	Q	I^2^	T^2^	% variance due to artefacts
Motivational	32	19,475	0.34	0.37	0.27;0.40	0.11;0.62	0.01;0.62	509.44***	93.91%	0.03	7%
Job satisfaction	13	6580	0.28	0.34	0.18;0.37	0.02;0.66	− 0.19;0.65	253.27***	95.26%	0.05	6%
Engagement	6	2634	0.35	0.34	0.24;0.46	0.23;0.44	0.12;0.55	17.90**	72.07%	0.01	37%
Work motivation	6	5520	0.40	0.43	0.33;0.47	0.32;0.54	0.20;0.58	31.75***	84.25%	0.01	19%
Affective commitment	5	4592	0.36	0.34	− 0.05;0.67	0.07;0.61	− 0.37;0.82	150.29***	97.34%	0.05	4%
Self-efficacy	2	149	0.54	0.39	− 1;1	− 0.1;0.78	− 1;1	14.37***	93.04%	0.49	20%
Self and career evaluation	9	2070	0.19	0.11	0.04;0.33	− 0.20;0.42	− 0.25;0.56	63.85***	87.47%	0.18	15%
Attitudes toward retirement	6	1519	0.08	0.03	− 0.08;0.24	− 0.17;0,23	− 0.27;0.42	22.48***	77.76%	0.02	27%
Career success	2	436	0.36	0.34	− 0.60;0.89	0.27;0.40	− 0.80;0.95	1.45	30.92%	0.01	53%
Self-control	2	115	0.32	0.32	− 1;1	0.15;0.48	− 1;1	–	–	–	–
Personal	15	6102	0.28	0.18	0.15;0.40	− 0.27;0.64	− 0.31;0.71	421.62***	96.68%	0.08	4%
Wellbeing	10	3697	0.36	0.32	0.22;0.49	− 0.05;0.68	− 0.18;0.73	186.77***	95.18%	0.06	6%
Health	3	2049	− 0.04	− 0.06	− 0.27;0.17	− 0.12;0.01	− 0.35;0.28	5.26	61.97%	0	66%
Job strain	2	356	− 0.28	− 0.26	− 0.99;0.96	− 0.54;0.02	− 1;1	10.65***	90.61%	0.06	19%
Relational	7	1975	0.29	0.29	0.23;0.35	0.27;0.31	0.20;0.38	7.30	17.78%	0	97%
Extra role behaviours	2	918	0.15	0.15	− 0.56;0.73	0.07;0.23	− 0.79;0.88	3.44	70.96%	0.01	68%

K-number of research; N-total population; $\overline{r}$-correlation; $\overline{r}$-sample size-weighted correlation; 95% CI-the confidence interval around the mean sample-weighted correlation; 95% CR-credibility intervals calculated using $\overline{r}$ and standard deviation; 95% PI-prediction intervals; Q-chi-square test for homogeneity of population correlations across studies; I^2^-percent of true heterogeneity; T^2^–the variance of the true effect sizes

* *p* < 0.05

** *p* < 0.01

*** *p *< 0.001

#### Motivational outcomes

The meta-analysis of relationships between generativity and motivational outcomes involved thirty-two independent samples. Thirteen samples concerned job satisfaction, six concerned work motivation and engagement, five concerned affective commitment, and two—self-efficacy. The total number of respondents in the samples was 19,475, giving an average of 609 respondents per sample. The sample size-weighted correlation ($\overline{r}$) for 32 samples was 0.37. The 95% credibility interval ranged from 0.11 to 0.62, thus the true score correlation was probably positive. The strongest positive relationship was found between generativity and work motivation $\overline{r}$ = 0.43 and self-efficacy $\overline{r}$ = 0.39. The corrected correlations for job satisfaction and affective commitment were 0.34. These results support hypothesis H1.

#### Self and career evaluation outcomes

The relationship between generativity and self and career evaluation outcomes was analysed using eight independent samples. Six samples concerned attitudes toward retirement, and two—self-control and career success. The total number of participants was 2,070 (230 per sample on average). The sample size-weighted correlation was 0.11. Both the 95% credibility and predictive interval include zero, which indicates weak questionable dependencies between self and career evaluation outcomes in general. The strongest positive relationships occurred between generativity and career success $\overline{r}$ = 0.34 and self-control $\overline{r}$ = 0.32. However, no relationship was obtained between generativity and attitudes toward retirement $\overline{r}$ = 0.03. It implies that in organisations that promote generativity, employees have a greater sense of career success and self-control, but they do not have more positive attitudes toward retirement. Thus, hypothesis H2 was partially supported.

#### Personal outcomes

The relationship between generativity and personal outcomes was assessed using fifteen independent samples. The total number of participants was 6,102 (47 per study on average). The sample size-weighted correlation was 0.18, and the 95% credibility and predictive interval include zero. The relationships between generativity and personal outcomes varied considerably depending on the component. Job strain was moderately and negatively associated with generativity $\overline{r}$ = − 0.26, well-being was associated moderately and positively $\overline{r}$ = 0.32, but for health, the relationship was low ($\overline{r}$ = 0.06). Thus, generativity has a positive effect on personal outcomes, excluding health, which partially confirms hypothesis H3.

#### Relational outcomes

The relationship between generativity and relational outcomes (positive relations with others) was examined empirically using seven independent samples. The total number of participants was 1,975 (282 per study on average). After correcting for artefacts, the relationship was 0.29, and the 95% confidence, credibility, and predictive intervals did not include zero. Thus, hypothesis H4 was supported.

#### Extra-role behaviour outcomes

A relationship between generativity and extra-role behaviours was examined empirically using seven independent samples. The total number of participants was 1,975 (282 per study on average). After correcting for artefacts, the relationship was (0.29), and the 95% confidence, credibility and predictive intervals did not include zero. Thus, hypothesis H5 was supported.

### Moderating effects of the measurement method and cultural contexts

Because statistical artefacts alone did not explain a significant portion of the variance in correlations in our analysis, we tested three categorial moderators (the measurement method, cultural context and age) to see if they could differentiate the tested relationships (Table 3). Extra-role behaviour was omitted because of the small sample (k = 2).

**Table 3:** Analysis of moderators of relationships between generativity and outcomes

Variable	K	N	*r*	$\overline{r}$	95% CI	95% CR	95% PI	Q	I^2^	T^2^	% variance due to artefacts
*Motivational*											
LGS	12	5890	0.42	0.41	0.29;0.41	0.18;0.63	0.07;0.67	316.70***	84.63%	0.03	7%
MWS	5	2157	0.26	0.35	0.29;0.41	0.32;0.37	0.27;0.43	4.56	12.33%	0	91%
GS	4	1349	0.17	0.18	− 0.04;0.37	− 0.03;0.39	− 0.31;0.58	21.01***	85.72%	0.02	19%
Others	5	1443	0.16	0.16	0.10;0.22	0.09;0.24	0.10;0.22	2.86	0	0	35%
North America	17	13,610	0.37	0.40	0.28;0.45	0.15;0.64	0.03;0.63	323.03***	95.05%	0.03	6%
Europe	14	5525	0.32	0.31	0.22;0.41	0.11;0.51	0.04;0.55	90.36***	85.61%	0.13	17%
Age < 55	18	6190	0.33	0.27	0.24;0.41	0.01;0.54	0.00;0.59	251.91***	92.85%	0.16	12%
Age > 55	14	13,285	0.36	0.40	0.24;0.46	0.15;0.65	− 0.06;0.66	253.75***	95.27%	0.19	4%
*Self and career evaluation*											
LGS	3	197	0.37	0.37	0.14;0.56	0.20;0.53	0.14;0.56	1.19	0%	0	98%
MWS	2	503	0.27	0.29	− 0.55;0.83	0.28;0.30	− 0.76;0.92	1.86	46.09%	0.07	99%
GS	2	900	0.04	0.02	− 0.89;0.91	− 0.18;0.21	− 0.98;0.99	11.25***	91.11%	0.02	18%
Others	2	470	0.02	− 0.01	− 0.81;0.82	− 0.10;0.08	− 0.94;0.94	3.01	66.78%	0.01	66%
North America	4	958	0.19	0.17	− 0.11;0.46	− 0.15;0.49	− 0.49;0.73	30.99***	90.32%	0.04	13%
Europe	4	722	0.20	0.02	− 0.22;0.56	− 0.27;0.29	− 0.58;0.79	21.07***	85.76%	0.06	20%
Age < 55	4	982	0.21	0.05	− 0.21;0.56	− 0.23;0.33	− 0.51;0.76	25.54***	88.26%	0.21	16%
Age > 55	5	1088	0.18	0.16	− 0.03;0.37	− 0.16;0.44	− 0.36–0.63	31.33***	87.23%	0.19	16%
*Personal*											
LGS	8	2938	0.40	0.38	0.24;0.54	0.07;0.69	− 0.10;0.74	114.36***	93.88%	0.04	8%
MWS	4	2649	− 0.03	− 0.05	− 0.14;0.09	− 0.12;0.03	− 0.23;0.18	7.81*	61.59%	0.01	51%
Others	3	515	0.22	0.27	0.13;0.40	0.16;0.38	0.13;0.40	1.20	0	0	98%
North America	5	866	0.33	0.29	0.02;0.56	0.16;0.38	− 0.28;0.74	29.10***	86,25%	0.04	20%
Europe	6	4103	0.12	0.06	− 0.05;0.28	− 0.23;0;35	− 0.22;0.71	102.64***	94.15%	0.03	7%
Asia	3	1133	0.54	0.55	0.35;0.69	0.48;0.61	0.23;0.75	4.96*	59.64%	0	52%
Age < 55	13	5286	0.31	0.23	0.18;0.43	− 0.15;0.62	− 0.23;0.70	284.89***	95.79%	0.25	5%
Age > 55	2	816	0.18	0.21	− 0.69;0.84	0.12;0.30	− 0.90;0.95	3.65	72.59%	0.10	52%
*Relational*											
GS	3	1156	0.29	0.22	0.24;0.34	0.04;0,41	0.24;0.34	0.48	0	0	20%
Others	4	1495	0.33	0.22	0.11;0.52	0.19;0.24	− 0.07;0.64	6.74	55.50%	0.01	93%
North America	3	1306	0.38	0.23	0.01;0.66	0.19;0.26	− 0.22;0.77	3.88	48.41%	0.01	87%
Europe	4	1345	0.28	0.21	0.24;0.32	0.04;0.38	0.24;0.32	1.16	0	0	16%

*LGS* Loyola Generativity Scale; *MWS* Meaning of Work Scale; *GS* Generativity Striving; other abbreviations—as in Table 2

* *p* < 0.05

** *p* < 0.01

***p < 0.001

#### Measurement methods

Generativity was measured using various tools, the most common of which were: LGS–23 times, MWS—11 times, and GS—9 times. When using the LGS then the MWS, the results showed the strongest effect between generativity and motivational outcomes (z = 2.786; *p* < 0.01) and personal outcomes (z = 16.790; *p* < 0.001), but not self and career evaluation outcomes (z = 1.062; n.s.). The lowest relationships between the variables were obtained for measuring generativity using the GS and other measuring tools. No differences between the results after taking into account the measurement tools were demonstrated for the relational dimension.

Our meta-regression tested the multivariate effect of the moderator variables (Table 4). The results revealed that our moderators explained 67% of the variance in the generativity-self and career evaluation outcomes and 32% in the generativity-motivational outcomes. We found no support for the moderator relation regarding personal and relational outcomes.

**Table 4:** Regression of correlations on the moderator

	Motivational			Self and career evaluation			Personal			Relational		
	*B*	*SE*	*Z-value*	*B*	*SE*	*Z-value*	*B*	*SE*	*Z-value*	*B*	*SE*	*Z-value*
Intercept	0.54	0.05	10.47***	0.52	0.12	4.38***	0.39	0.13	2.90**	0.27	0.09	3.07**
Measurement method	− 0.10	0.02	− 4.18***	− 0.14	0.04	− 3.23***	− 0.06	0.06	− 0.90	0.03	0.06	0.42
Sum of squares (Q) (model)	17.59***			10.41***			0.81			0.17		
R^2^	0.32			0.67			0.07			0.03		
Intercept	0.49	0.08	5.96***	0.21	0.19	1.08	0.12	0.20	0.60	0.50	0.13	3.75***
Cultural context	− 0.09	0.05	− 1.79	− 0.01	0.11	− 0.09	0.09	0.10	0.91	− 0.10	0.07	− 1.50
Sum of squares (Q) (model)	3.22			0.01			0.84			2.25		
R^2^	0.07			0.01			0.07			0.31		
Intercept	0.33	0.10	3.30***	0.23	0.24	0.96	0.12	0.20	0.60	–	–	–
Age differentiations	0.02	0.06	0.30	− 0.03	0.18	− 0.18	0.09	0.10	0.91	–	–	–
Sum of squares (Q) (model)	0.09			0.03			0.84			–		
R^2^	0.02			0.05			0.07			**–**		

* *p* < 0.05

** *p* < 0.01

*** *p* < 0.001

#### Cultural context

The studies were conducted in North America (*k* = 29), Europe (*k* = 28) and Asia (*k* = 3). Stronger motivational (z = 6.462; *p* < 0.001), self and career evaluation (z = 3.072; *p* < 0.001) and personal (z = 6.368; *p* < 0.001) outcomes of generativity occurred among respondents in North America than Europe. However, in the case of personal outcomes, stronger relationships between the variables were obtained in the Asian cultural context than both North America (z = 7.074; *p* < 0.001) and Europe (z = 16.617; *p* < 0.001). Our meta-regression analysis showed that the cultural context as moderator explains 31% of the variance in relational outcomes but only 7% in both motivational and personal outcomes.

#### Age differentiations

Among the available samples, we located 21 samples in which surveys were conducted among workers over 55 years of age. In contrast, 44 samples were conducted among workers under 55 years of age. The analysis show that older workers have a statistically stronger activating motivational component (job satisfaction, work engagement, work motivation, affective commitment, self-efficacy) than younger workers—under 55 years old (z = -2.213; *p* < 0.05). Conversely, workers under 55 years of age have a higher sense of personal well-being than older workers (z = 3.678; *p* < 0.001). No differences were identified in the self and career evaluation dimension. An additional meta-regression analysis did not indicate significant linear relationships in the moderators tested.

## Conclusions and discussion

In line with Eriksonian developmental theory (Erikson 1982), successful ageing theory (SOC), and the socioemotional selectivity theory (SST), and taking into consideration research findings about generativity in the work context from the last three decades, we proposed a conceptual model of outcomes of generativity at work. In contrast to the last meta-analysis of generativity prepared by Doerwald et al. (2021), we decided not to concentrate on the antecedent of generative behaviour but on the generativity work-related outcomes, and our results are broader in the work-related context. Our findings improve understanding of how generativity relates to individual work outcomes and proposes an alternative way to operationalise work outcomes in this context. Our conceptual model groups constructs into five categories that cover not only productivity and career outcomes but also individual work-related attitudes such as commitment and motivation, and even pro-social and pro-ecological behaviour. This line of operationalisation let us include more studies than in other meta-analyses (Allen et al. 2004; Kooij et al. 2011; Ghosh and Reio 2013; Rudolph et al. 2018; Allan et al. 2019). As a result, our meta-analysis examines 65 independent samples (*k*) that includes 30,540 individuals (*n*). In Doerwald et al.’s (2021) meta-analysis, the outcome groups are not the same as in our approach. They distinguished three groups of outcomes: motivational (motivation, self-efficacy), well-being (e.g. job satisfaction, job strain, life satisfaction, affective commitment) and career-related (e.g. career satisfaction, motivation to continue working, mentoring relationship quality). Thus, our results are not directly comparable, although for some individual outcomes, our results are similar.

Our results support most of the hypotheses—the findings confirm positive relationships between generativity and:

- Motivational outcomes (job satisfaction, engagement, work motivation, affective commitment, self-efficacy), which supports H1,
- Two of three self and career evaluation outcomes (career success and self-control), which partially supports H2,
- One of three personal outcomes (well-being and job strain), which partially supports H3,
- Relational outcomes, which supports H4,
- Extra-role behaviour, which supports H5.

Hypotheses H2 and H3 are partially supported—generativity was not found to be significantly related to employees’ health, as well as—attitudes toward retirement. In the context of health it should be noted that in the analysed studies respondents’ self-assessments of health and not objective measures are placed. Moreover, the assessments concerned general health without providing any details of physical health or mental health. The correlation between generativity and health reported by the analysed studies was mostly positive (de Lange et al. 2010; Homan et al. 2020; Melo 2008; Newton et al. 2020), although sometimes negative (Kooij et al. 2013a). With regard to health, it would be interesting to know whether the pandemic will make organisations more willing to implement generativity practices or whether it will discourage their use and diminish their role. Kooij (2020) presumes that older workers may be more affected by the pandemic than younger workers, and Ayalon et al. (2021) add that they are labelled vulnerable and at risk of COVID-19. Like health, the third component of personal outcomes, job strain, was moderately and negatively associated with generativity. Garcia et al. (2018) and Lan et al. (2021) presented similar results regarding job strain being indicated by high job stress combined with low job satisfaction.

In line with socioemotional selectivity theory, as workers age, their limited future time perspective strengthens relation between their generativity motives and emotional outcomes. The salience of emotionally meaningful goals increased (i.e., emotion regulation and generativity). Thus, older workers should be more motivated by characteristics of their job that are socially and emotionally satisfying (e.g., having autonomy over their own work, having positive social interactions with co-workers) and less motivated by aspects of their job that revolve around accumulating resources (e.g., salary, job training) (Cavanagh et al. 2020), and this should affect job satisfaction of older workers and consequently—their occupational well-being. As suggested by SOC theory, by using the strategies of selection, optimization, and compensation older workers maintain their performance and minimizing loss during aging. Employees who use SOC strategies perceive themselves as efficient and effective in pursuing their work goals, and their emotional and personal outcomes, e. g. job satisfaction, engagement, occupational well-being should improve. The use of SOC strategies may also positively impact on self and career evaluation outcomes, such as career success, intention to remain in (bridge) employment (Moghimi et al. 2017). Thus, older workers not always are more likely to select orientation towards compensation—employees with higher generativity values reported even higher optimization orientation than younger workers. In particular, emotion-oriented values, generative and autonomy goals are more important for older workers (Hertel et al. 2013). It is important in the mentoring as well as leadership context. Older leaders with higher generativity had higher levels of leadership success than leaders lower in generativity (Rudolf et al. 2018).

In our analysis we considered also the role of three moderators. The generativity measurement method, and age was also analysed by Doerwald et al. (2021), while the inclusion of the cultural context is our new approach. Regarding the measurement method, we confirmed Doerwald et al.’s (2021) findings that the Loyola Generativity Scale is the most popular, but in the job context, two other measurement methods—the Meaning of Work Scale and Generative Striving—are often used, too. These three methods measure generativity concern; however, studies that concern behavioural approach are rare in the job context (McAdams and de St. Aubin 1992; Stamov-Roßnagel and Biemann 2012). For most of the analysed outcomes, excluding relational ones, measurement method has the moderate effect, and the strongest is for self and career evaluation and emotional outcomes.

The cultural context helps us to shed light on possible cultural variations in generativity in the work context. For North America samples—more than their European—the strongest motivational, self and career evaluation and personal outcomes are observed, while in the case of personal outcomes the strongest relationship was obtained in the Asian cultural context. That could be interpreted as support for the cultural differences widely express by Hofer et al. (2014). It suggests that cultural contexts, including traditions and family relationships, can contribute to the expression of generativity (Rubinstein et al. 2015), while altruistic goal attainment mediated the association between generative concern and positive emotion (Au et al. 2020). An alternative explanation for these differences could also be the fact that representatives of eastern cultures (e.g. Japan, China) have a tendency to choose the midpoints in the scales, while representatives of Anglo-Saxon countries have a tendency to choose the extreme values (Shimazu et al., 2010).

For the age variable, analyses showed that older workers have higher levels of motivational outcomes and lower levels of personal outcomes (wellbeing and health) then younger workers. This is consistent with both Doerwald et al.'s (2021) meta-analysis on predictors and effects of generativity and meta-analyses on workers' age and work attitudes (Ng and Feldman 2010).

## Limitations

In our study we used the approach proposed by McAdams et al. (1993), whereby generativity can be conceptualized as a broad theoretical construct with various, interrelated facets that might best be measured in different ways. The purpose of the study was to identify individual outcomes of generativity based on a review of studies on this topic. However, the variety of research methods used by their authors affected the comparability of their findings, making it difficult to create a coherent picture of generativity.

Another limitation in interpreting the results of our study is the use of self-reported data. Such data impair the objectivity of assessments of the analysed features, especially in relation to outcomes, but also to generativity measurements. This way of analysis was a consequence of the approach adopted in the papers identified in this area. Studying the effects of generativity using the perspective of not only one group of respondents, be it employers, workers, etc., would be better. The optimal approach should consider employees’ assessments, as well as the opinions of their co-workers, subordinates, immediate superiors, and higher-level managers. Such feedback would help create a more complete picture of generativity. Meanwhile, studies in this context are rare. For example, the literature search conducted in 2020 generated only two works on the relationship between generativity and outcomes measured from the perspective of HRM. Carmeli et al. (2016) assessed the relationship between caring relationships and strategic adaptability, finding it to be mediated by generativity, and Carmeli and Dothan (2017) demonstrated the power of generativity in the workplace as a key source of learning in innovation management processes.

It is important to note several other limitations in procedure of our meta-analysis. First, many of the relationships estimated involve a small number of studies; hence, it should be recognised that second-order sampling error poses a threat to the validity of our results (Hunter and Schmidt 2004). Second, we were unable to search any other moderators because sufficient data were unavailable. Although this limitation is common in meta-analytic research, the variability in effect sizes suggested the presence of moderators for a number of relationships with generativity (e.g., job satisfaction, well-being) that we were unable to investigate. Third, the cross-sectional nature of most studies of generativity precludes making causal inferences, even though the theoretical model on which our meta-analysis was based, i.e., socioemotional selectivity theory (SST) (Carstensen et al. 1999), implies directional relationships. We echo the call of many generativity researchers regarding the need for longitudinal research designs to better address the causal nature of relationships that involve the effects of generativity. Finally, although meta-analytically derived effect sizes are often designated as population parameters, it is important to recognise that the observed relationships do not represent a defined population per se. Because they are based on data aggregated from a large number of samples, meta-analytic results are more dependable than those based on smaller samples, although they are not true population parameters and should not be interpreted as such.
